# Maximising Grip on Deception and Disguise: Expert Sports Performance During Competitive Interactions

**DOI:** 10.1186/s40798-022-00441-y

**Published:** 2022-04-08

**Authors:** Harry Ramsey, Matt Dicks, Lorraine Hope, Vasu Reddy

**Affiliations:** 1 Department of Psychology, University of Portsmouth, King Henry Building, King Henry I Street, Portsmouth, PO1 2DY UK; 2 School of Sport, Health and Exercise Science, University of Portsmouth, Portsmouth, UK

**Keywords:** Maximum grip, Misleading affordance, Continuum of contact, Deception, Disguise, Anticipation

## Abstract

Expert performers in fast-ball and combat sports continuously interact with their opponents and, if they are to be successful, adapt behaviour in order to *gain an advantage*. For example, disguise and deception are recognised as skilful behaviours that are employed to disrupt an opponent’s ability to successfully anticipate their actions. We contend that such skilled behaviour unfolds during the interaction between opposing players, yet typical research approaches omit and/or artificially script these interactions. To promote the study of skilled behaviour as it emerges during competitive interactions, we offer an account informed by contemporary ecological perspectives for shaping investigation into how deception and disguise can be used to gain an advantage over an opponent and the challenges it poses to anticipation. We propose that each player attempts to develop *maximum grip* on the interaction through exploiting information across multiple timescales to position themselves as to facilitate openness to relevant affordances. The act of deception can be understood as offering a *misleading affordance* that an opponent is invited to act on, imposing a significant challenge to an opponent’s ability to attain grip by manipulating the information available. Grounded in our ecological perspective, we emphasise the need for future investigation into: (1) the role of disguise for disrupting anticipation; (2) how deception can be employed to gain an advantage by manipulating information on multiple timescales, before detailing; (3) how opposing performers go *beyond merely exploiting information and actively elicit information* to deal with deception and disguise during an interaction.

## Key Points

- We offer an ecological account to promote the need to study deceptive behaviour in sport using research designs that facilitates performers to interact in order to enhance understanding about how experts gain an advantage.
- Future research focussed on the relationship between deception and context would inform understanding of how performers gain an advantage by being responsive to information on shorter and longer timescales concurrently in order to maximise their grip on the interaction.
- Research approaches that maintain the interaction between individuals permit investigation into how a performer actively influences an opponent’s actions to reduce uncertainty and enhance their anticipation performance by actively eliciting information available to be perceived.

## Introduction

Expert sports performers [1–3], continuously interact with their opponents and, if they are to be successful, adapt behaviour in order to *gain an advantage*. Performers learn to successfully anticipate others actions, utilising information from an opponent’s kinematics [1] and contextual information from the situation at large [4]. Additionally, evidence has highlighted that performers use disguise and deception [5] to challenge opponents during interactions [6]. Deception involves coordinating an action to convey a false intention, whereas disguise involves coordinated action that conceals veridical information from an opponent until as late as possible [7, 8]. However, research approaches investigating anticipation often reflect methodological tendencies which aim to maximise experimental control likely at the expense of sufficiently representing the sport situations that the research is intended to generalise to. For instance, video-based designs separate the interaction between performers [9], and to date, both video and in situ studies have artificially scripted the interaction [10]. While these methods offer experimental control, they limit understanding of the perception–action abilities of skilled performers during a series of complex unfolding interpersonal interactions [10].

In this opinion paper, we contend that new lines of research, which aim to study skilled interactions have the potential to contribute theoretical and practical knowledge of expertise [3, 11, 12]. To promote these lines of enquiry, we outline an account informed by contemporary ecological perspectives [13–15] for framing how deception and disguise can be used to gain an advantage. Drawing on this account, we develop a rationale for future work to investigate: (1) the prevalence and influence of disguise on anticipation; (2) how performers manipulate information across multiple timescales to deceive; and (3) how players go beyond merely exploiting information and actively elicit information to successfully anticipate an opponent.

## *Maximum Grip* on Disguise and Deception: An Ecological Perspective

### Anticipation in Competitive Interpersonal Interactions

Ecological approaches are grounded in the proposal that a person’s behaviour is visually guided by the perception of *affordances* [16]. That is, the opportunities for action that an environment offers a performer relative to their abilities [17]. During interactions, affordances evolve relationally^1^ between performers—how one acts modulates how the other acts [3, 11]. Therefore, interpersonal interactions can be understood as a toing and froing between perceiving and acting with the aim of influencing one’s own and stifling opponents’ available affordances to maximise opportunities for success [3, 10]. Fundamental to the ecological approach considered in this paper, is the recognition that skilled performers actively explore and interact with an opponent [21], perceiving and adapting with the aim of developing *maximum grip* on the situation [3, 13]. Maximum grip considers how performers exploit information across multiple timescales (contextual information) to position themselves to facilitate openness to relevant affordances [3, 13]. Openness to affordances can be viewed as means to maintaining adaptability whereby the performer is sufficiently prepared to adapt to the changing affordances [13]. Importantly, maximising grip requires sufficient *epistemic contact* with the interaction. Withagen [14] proposed a *continuum of contact*, inspired by Gibson’s [16], p. 239] proposal that perception is a way of “keeping-in-touch with the world”. The more useful the information exploited, the greater degree of contact a performer has with the environment. Drawing on Rietveld and colleagues [13, 18], we consider the affordances available to an individual performer in a given situation as the *field of affordances*. Affordances in an individual’s field invite behaviour, with some inviting behaviour to a greater extent than others [22]. As Rietveld and Kiverstein [18], p. 342] succinctly emphasise “typically, it will only be those affordances that will (appear to) improve an individual’s *grip* [emphasis added] on the particular situation that will invite or solicit an individual’s action”.

Performers often need to anticipate others’ actions due to extreme spatiotemporal demands [6, 23] in close proximity interactions such as combat sports [10], 1-versus-1 defender–attacker interactions [1, 24, 25], and distal interactions such as football penalty kicks [26]. These situations demand that performers exploit earlier, non-specifying information that correlates with an event outcome but is not specific to it^2^ [14, 29]. Extant literature has investigated how anticipation is supported by two primary forms of information: (1) information from opponents’ *kinematics* and (2) *contextual* information over longer timescales. Use of information from kinematics is exemplified by a goalkeeper anticipating kick direction from the movements of the opponent during a penalty kick [6]. Whereas contextual information reflects the circumstances preceding and surrounding an interaction [30], denoted, for example, by an opponent’s behavioural pattern generated over a sequence of events [31]. Contextual information is available prior to and during an interaction, existing on a longer timescale than kinematic and ball-flight information that develops during a given interaction between two opposing performers. Therefore, expert performers must be attuned to information on multiple timescales to develop sufficient contact and maximise their grip on the interaction [3, 10].

Findings indicate that experts are better able to utilise information from both kinematics [32, 33]^3^ and context [30] to attain contact, develop grip, and guide successful anticipation. Furthermore, given the reciprocal nature of perception and action, one performer’s actions influence the actions of an opponent [11]. Thus, expert performers actively influence an opponent’s actions, reducing uncertainty in the upcoming event. This adaptive process is utilised by both performers, as the engaged defender and attacker aim to maximise their grip on the interaction [3, 10].

### The Role of Disguise and Deception for Maximising Grip

Performers use disguise and deception to challenge an opponent’s ability to develop grip and accurately anticipate their actions. A well disguised movement conceals veridical information from an opponent until as late as possible in their action to maximise ambiguity [7, 8], and limit opponents’ ability to improve their grip on the interaction. In the football penalty kick, for example, the penalty taker can control their actions in a manner that affords multiple kick directions until late in their action [5]. This challenges the goalkeeper to delay their response and act on the limits of, or even outside of, their action capabilities in order to exploit useful information from the kicker’s actions and ball-flight. Consequently, the goalkeeper may not have sufficient time to dive and intercept the ball [34, 35]. Alternatively, the goalkeeper can initiate their dive with sufficient time to intercept the ball, but the kinematic information available at that time point may not reveal the direction of the kick.

**Fig. 1:**
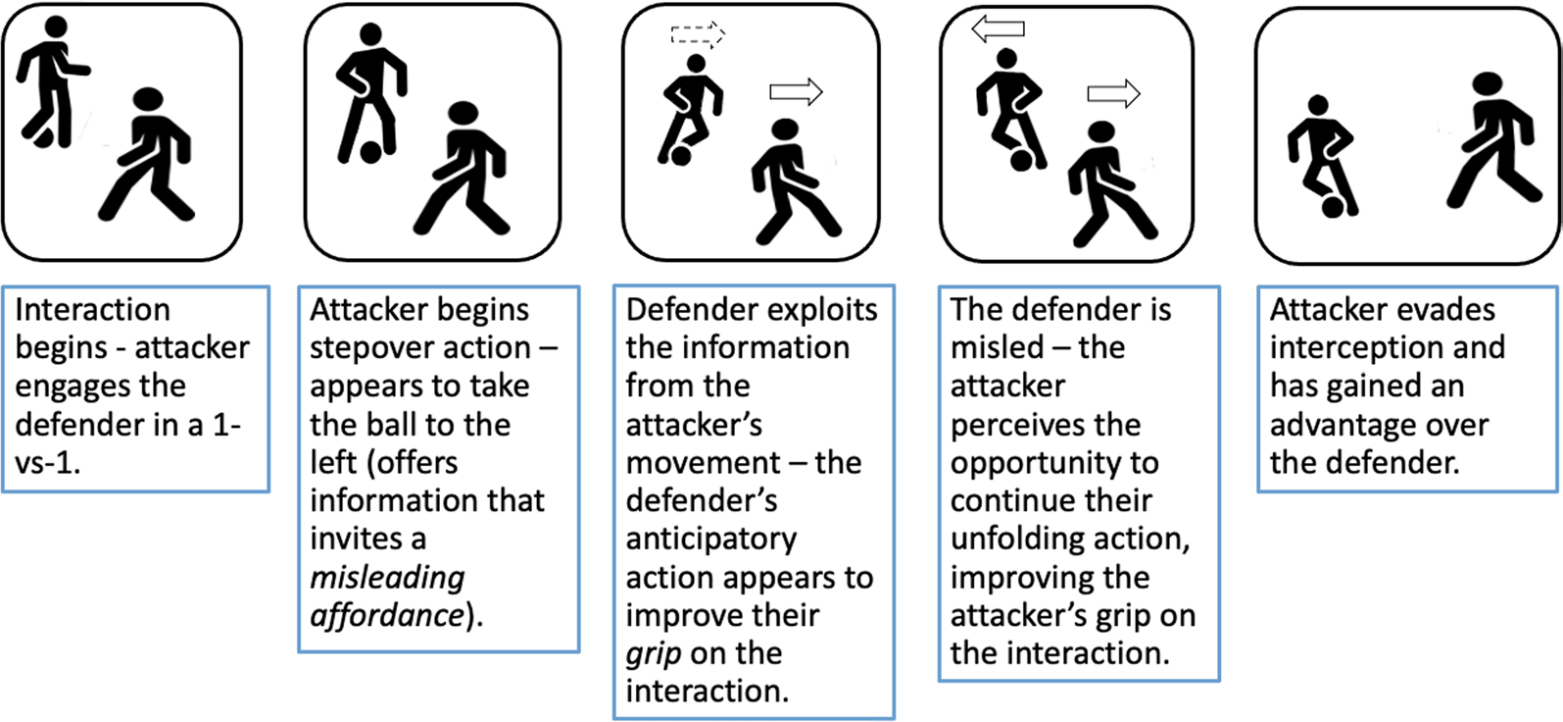
Phases of a 1-versus-1 attacker-defender interaction in Association Football (soccer) with the example of an attacker using a stepover to deceive the defender and mislead them from their true intention (football player icons made by Freepik from www.flaticon.com [41, 42])

Deception involves coordinating an action to convey a false intention, before adapting later in the movement to perform the actually intended action [7, 8]. Recently, Kimmel and Rogler [3] suggested that affordances during competitive interaction can be misleading, and therefore, deception can be viewed as offering information that induces a *misleading affordance*, falsely offering grip to an opponent. When facing a deceptive action, a defender exploits information from the attacker’s unfolding action. This information appears to offer the defender grip, inviting an interceptive response. As the interaction develops, the inviting affordance which solicited the defender’s action may actually *mislead* them from the attacker’s intention, reducing the defender’s grip on the situation, shifting the advantage to the attacker. Figure 1 depicts this unfolding process with the example of a stepover in Association Football (soccer). Deceptive actions mimic, or exaggerate [24] information that can be exploited from non-deceptive actions, encouraging early but misdirected anticipation [36]. That is, performers can invite an opponent to engage in a given action which creates instability in the interaction and opens up affordances that can be exploited to gain an advantage. A common sporting example of this is a misdirected pass in basketball, whereby a player appears as if they are going to pass one way, inviting early anticipation that creates an affordance to pass to another teammate [37]. The efficacy of disguise and deception is supported by a body of research that shows performers are less successful at anticipating such actions compared to non-deception [1, 6, 7, 34, 37–40].

## Novel Considerations for the Study of Disguise and Deception in Interaction

Although significant advances have been made in the study of deception and disguise, several important questions remain unexplored. In the following section, we overview three emergent gaps, and for each gap identified we propose three future research directions adopting the ecological approach put forward.

### Experts Frequently Disguise Their Actions

Extant literature has primarily compared deceptive and non-deceptive actions [7] or merged deceptive and disguised actions into one category [38], whereby video stimuli that reflect only the best non-deceptive and deceptive actions are included in a study [43]. While this approach permits rigorous comparison between deception and non-deception, it has meant that investigation into the effect of disguise on anticipation has been neglected because disguised actions that neither represent non-deception nor deception are not studied. This is problematic as it appears intuitive to consider that disguise is replete within experts’ actions and likely replete within their typical actions in order to limit opponents’ ability to develop grip. This is evidenced by the recurrent finding that both skilled and less-skilled players demonstrate success that is often significantly above chance levels when anticipating non-deception [7, 38, 39, 44–47]. This emphasises the need for experts to regularly employ disguise within their actions if they are to succeed against expert opponents.

Research by Esteves and colleagues [25] found that skilled attackers are better able to conceal information when executing a basketball drive by initiating their attack with a smaller forward movement of their advanced foot, despite no instruction to disguise their actions. The authors inferred skilled players had learned to conceal information in their typical movements. Moreover, in an unscripted study, Hunter [26] and colleagues found that penalty takers’ run-up angle was neutral in 64% of trials, categorised by a run-up that implied a shot “down the centre of the goal” (p. 2754). However, kickers shot in multiple directions with a neutral run-up angle, supporting previous research that has found run-up angle to be largely incongruent with kick direction [5, 48, 49]. Therefore, skilled players appear to disguise their actions, affording themselves multiple options, making their actions ambiguous by limiting information for goalkeepers. As a result, goalkeepers are forced to delay their response and act outside of their action capabilities [34], relinquishing their grip on the interaction as they will not have sufficient time to respond in the right place at the right time [50].

The studies [25, 26] considered in the previous paragraph suggest that disguise potentially reflects the most common action performed and reciprocally perceived, yet investigation into this facet of expertise has been neglected. Following the approach of Triolet et al. [51], who analysed videos of expert tennis performers' anticipation during competition, an important avenue for future work is to determine the frequency and the nature of experts’ use of disguise across various unscripted interactive situations. Indeed, the first stage in Ericsson and Smith’s ([52], see also [53]) expert performance approach is to perform a task analysis to capture key performance components in situ during representative conditions. Whilst research has yet to clearly distinguish the kinematics of disguise from deceptive and non-deceptive actions, motion capture methods [48, 54] as well as recent developments in reliable systematic observational coding systems for interactive sport actions [55] present promising approaches to investigate disguise.

Additionally, studies may replicate the approach taken by Hunter et al. [26] to investigate the effectiveness of disguise for disrupting anticipation performance in comparison to non-deceptive and deceptive actions. Such studies would give insight into the extent to which disguise masks adaptive information, limiting the degree of contact that an opponent can attain to maximise their grip. One intuitive question is to explore how opponents balance the need to delay their actions to exploit the most useful information to develop contact, with the need to initiate their actions early to afford enough time to act in the right place at the right time. Finally, one avenue to investigate further is whether disguised actions are as susceptible to reduction in action accuracy as has been demonstrated with deceptive actions because of the constraints on coordination^4^ [56–58]. Future research may find that such constraints do not persist for disguised actions, affording players the opportunity to disrupt an opponent’s ability to develop grip whilst maintaining execution accuracy.

### The Role of Context in Successful Deception and Disguise

Little is known about the relationship between deception and disguise, and contextual information [8]. Researchers have often advocated for the importance of using context to facilitate anticipation performance [59, 60] and highlighted experts’ superior ability to exploit this information [61, 62]. Milazzo and colleagues [62] found that expert karate athletes were better able to detect that their opponent was employing the same attack every fourth action, displaying earlier and more accurate responses to the repeated action than were intermediate karate athletes. While these findings highlight experts’ superior ability to attune to contextual information, the scripted nature of the study limited the opportunity to study how actions emerge during an interactive dyad. In typical sports environments, experts are unlikely to predictably repeat actions in this manner, and if they do, it is likely they will adapt when they perceive that their opponent has attuned to it [10].

A consideration for future work is whether an attacker can manipulate context to their advantage by performing an action that is incongruent with a previous pattern [63, 64]. In a seminal study, Gray [31] observed that baseball batters’ swings were more accurate when facing fast pitches preceded by three fast pitches compared to fast pitches preceded by three slow pitches, highlighting that batters use patterns from the pitch sequence to control their swing. Building on Gray’s [31] finding, a batter may attune to the pitcher’s delivery pattern (e.g., consecutive fast deliveries) and anticipate the pattern to continue. Perceiving that the batter is attuned to this pattern, the pitcher can throw a different pitch (e.g., slower pitch) inviting the batter to initiate an erroneous action. In such scenarios, the pitcher maximises their grip on the interaction by manipulating information on a longer timescale to invite their opponent into a misdirected action. Therefore, it is not only that experts are unlikely to perform actions consecutively in a predictable manner, but also that they can, and likely do, deceive an opponent by manipulating context to offer an opponent a misleading affordance that falsely offers grip [3].

Performers who are capable of disguising their actions regularly can maximise their ability to deceive through manipulating context. Helm and colleagues [65] found that participants attributed a greater weighting to contextual information for guiding their judgements when the information from kinematics was ambiguous. Taking basketball as an example, a player may use disguise and make their movements ambiguous until as late in the movement as possible. The repeated use of disguise in similar situations on the court may lead an opponent to resort to using contextual information to anticipate [65]. That is, if the attacker had mainly passed to one court location, the defender may begin to anticipate this pattern [31, 36, 60, 66]. Therefore, future research should explore the extent to which performers whose actions are disguised limit perceivers to rely on contextual information to a greater degree, and therefore, are afforded the ability to manipulate context and elicit a misdirected response.

**Fig. 2:**
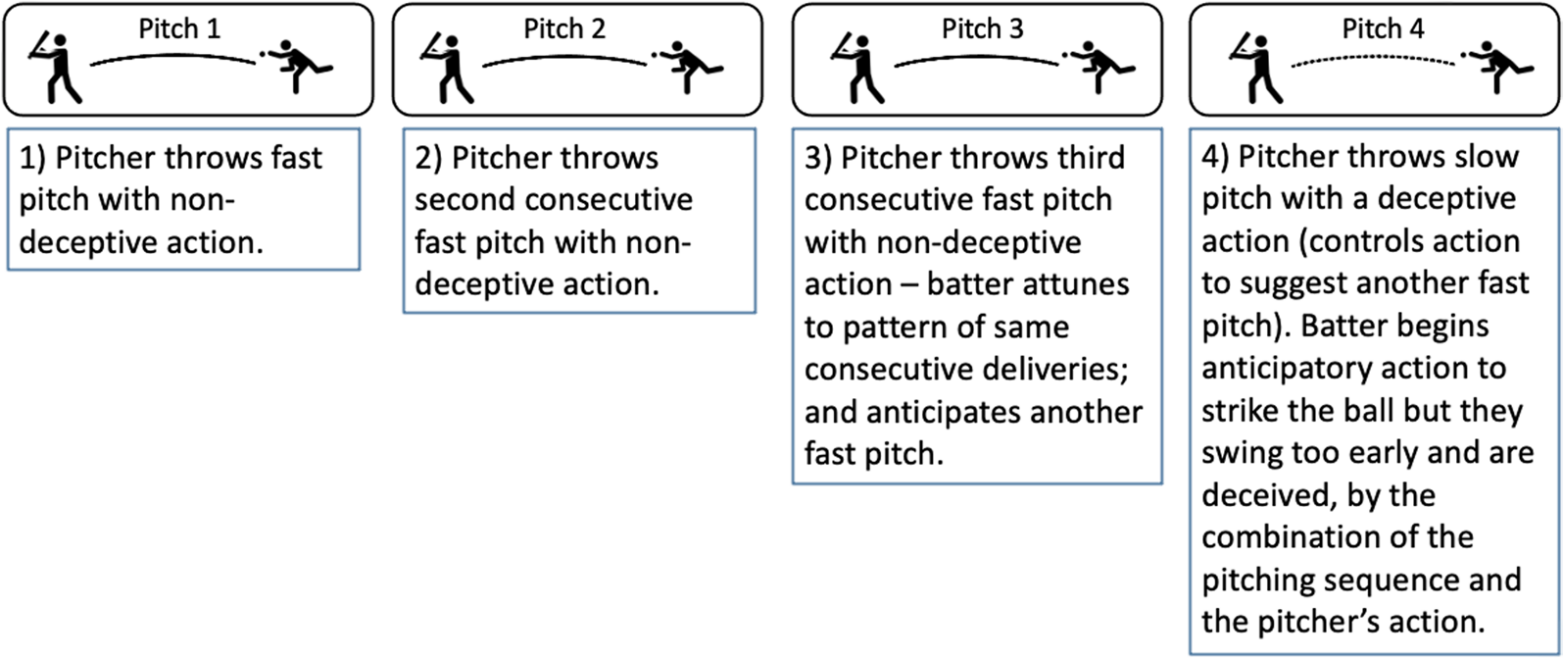
Baseball pitcher manipulating the sequence of their actions (information over a longer timescale) to maximise the likelihood of misdirecting a batter when they perform a deceptive action (information over a shorter timescale). Batter is deceived through the apparent congruency of pitch sequence and unfolding action of the pitcher in the fourth delivery (baseball player icons made by Freepik from www.flaticon.com [68, 69])

The manipulation of context may be combined with deception to maximise an opponent’s susceptibility to deception. Video and in situ designs have tended to use scripts, prescribing when deceptive actions occur and an equivalent number of deceptive and non-deceptive trials [7, 34, 39]. However, Hunter and colleagues’ [26] novel design gave players the choice of when to deceive and observed that deception was used in only 14% of trials. Importantly, this finding implies that the skill in performing successful deception may not only reside in how the action is performed [5] but also *when* deception is performed. That is, performers can manipulate both context and kinematics concurrently to deceive, falsely offering grip to an opponent by eliciting a misleading affordance, creating an opportunity for the deceiver to violate the pattern and gain an advantage. Figure 2 depicts an example of a performer manipulating the sequence of their actions to maximise their opportunity to mislead their opponent with a deceptive action. Future research focussed on the relationship between deception and context would inform understanding of how performers maximise grip and gain an advantage by being responsive to affordances on shorter and longer timescales concurrently, providing an exciting direction for future work [67].

Notably, one recent study investigated the relationship between deception and context [63]. Investigating the football stepover, Jackson and colleagues found that skilled performers’ susceptibility to deception was significantly greater when the direction of the deceptive fake aligned with the most probable action-outcome compared to judging deception when the probability information was balanced. This observation implies that successful deception involves deploying it in a context that enhances its likelihood of success. However, studies have found anticipatory behaviours recorded during video-based designs do not translate to anticipation under in situ conditions [34, 70, 71]. Additionally, Jackson et al. [63] manipulated situational probability information, which may only be available for structured situations such as the football penalty kick. Other forms of contextual information are more likely available during 1-versus-1 interactions, such as patterns generated over a series of events [31] or relationships between opponents’ positioning and action selection [72]. Therefore, whether these initial findings replicate in situ and with other sources of contextual information requires investigation. Specifically, future research should adopt in situ designs that require performers to coordinate an interceptive action and manipulate representative forms of contextual information to further develop transferable knowledge on the relationship between contextual information and susceptibility to deception.

### Active Interaction: Toward an Active Elicitation of Information for Dealing with Deception and Disguise

Research has investigated how skilled performers in defensive situations exploit the most veridical sources of kinematic and contextual information to guide their actions. However, underappreciated within the literature is the notion that defenders do not merely exploit information through eye and head movements, but they also have influence over eliciting information that is available to be perceived [3, 10]. That is, performers *actively* interact to influence an opponent’s action, which modulates the available kinematic and contextual information and therefore, the affordances available [3, 10, 73]. Studies of the penalty kick have shown that goalkeepers can successfully distract the penalty taker to reduce shot accuracy [74], manipulate their shot direction by positioning themselves marginally off-centre [75], and even influence shot direction with their mere presence [76]. Therefore, through interacting, performers can reduce the uncertainty about their opponent’s likely action, improving their grip on the interaction. Consequently, studies that do not permit an opponent to interact and influence the attacker likely moderate their ability to act successfully. Future research should therefore adopt in situ methods that allow performers to interact in order to investigate anticipation.

A question for future work is whether performers position themselves as to limit the number of affordances available to an opponent. For example, a defending player may block the line of pass to a teammate, position themselves between two opposition players to provide sufficient time to intercept a pass to either player, or encourage an opponent to engage in a less favourable 1-versus-1 interaction. These examples can be viewed as an attempt to disrupt an attacker’s ability to maintain an openness to affordances [3, 13], limiting their field of affordances and demanding they act on a less favourable affordance. This maximises the defender’s grip on the interaction as they are better aware of their opponent’s available affordances, making their actions more predictable and affording the defender the opportunity to be better prepared to intercept the attacker’s actions [13].

A further avenue for future investigation is to explore whether performers offer a specific affordance to the attacker, inviting them to perform a particular action. For example, expert tennis players reported that they positioned themselves on court to influence the serve their opponent performs, attempting to bluff them into performing a less favourable serve [77]. Accordingly, the anticipator becomes the deceiver; they set a trap, encouraging an opponent to act upon a misleading affordance and consequently, anticipate their actions with greater accuracy [3]. Therefore, performers go beyond merely exploiting information and *actively elicit information* to maximise grip on the interaction. In such interactions, the attacker is also the perceiver whereby they exploit information from the defender’s actions [25]. Skilled attackers are likely aware of a defender’s strategy to limit their options or offer a misleading affordance, and in response, attempt to employ disguise and deception themselves. Accordingly, skilled deception, whether from an attacker or defender, requires brinkmanship whereby performers act on the limits of their abilities in unstable regions in order to destabilise the interaction and gain an advantage [3, 10]. Important here is that both performers are perceiving and acting, both offering misleading affordances across multiple timescales to gain an advantage over the other simultaneously. This ongoing loop within a dyad, which is at the core of expertise in sport, is currently not well understood in the literature [26]. Experimental designs that permit performers to freely interact with their opponent [10, 26], coupled with the use of quantitative and qualitative measures [78] to investigate performers’ intentions over a series of interactions, would enable further understanding on how deceit and disguise are used to gain an advantage. Thus, the study of skilled human interactions in sport offers a fascinating area of potential for investigating skilful human interaction more generally.

## Conclusion

This paper offers an ecological approach to disguise and deception in sport and identifies important gaps in the literature. We have proposed that the skill of deception emerges during the interaction between two or more competing individuals both attempting to *maximise their grip* on the situation. The ecological approach frames the act of deceiving as offering a *misleading affordance* through manipulating the information available, limiting opponents’ ability to exploit useful information and improve their grip on the interaction. Applying this ecological perspective, we highlight the need to: (1) pursue in situ research that maintains the interaction to develop understanding of deception and investigate the extent to which experts’ typical actions are disguised; and (2) manipulate information that is representative of the specific sport situation sampled during experimental studies. Such methodological approaches enable investigating the extent that successful deception is not only dependent on coordination of action but also on timing when deception is used effectively. Additionally, by permitting the defender to freely interact with their opponent, researchers can investigate how performers go *beyond merely exploiting information and actively elicit information* through influencing their opponent to limit their available affordances to maximise their own grip on the interaction and become the deceiver themselves.

## Data Availability

Not applicable.

## Footnotes

1 Although we refer to the affordances available to a performer in a given situation (field of affordances [18]) as evolving relationally between interacting performers, we acknowledge that there is an ongoing debate in the wider ecological psychology literature. One school of thought proposes that affordances are dispositional characters of the environment [19], whilst another proposes that affordances are relational properties between a perceiver and their immediate environment [20]. There have also been attempts to reconcile these two positions, taking the position that affordances exist outside of the individual-environment interaction (landscape of affordances) but that the affordances available to a perceiver in their immediate environment (field of affordances) are relational [18].

2 During the execution of an action, performers can adapt their coordination through continual pick up of information [27], such as a player using momentary information on the ball relative to themselves in order to adjust the movements of their arms and hands to catch the ball [28]. However, for the purpose of the paper, we limit our discussion to the situations in which performers are required to initiate their action by acting on an affordance (opportunity for action) before specifying information is available, and thus, have exploited non-specifying information to support their action [14].

3 The paper by Travassos and colleagues [33] targeted decision-making behaviour. However, on inspection of their included studies, criteria considered, and measures analysed, it was apparent the analysis was assessing anticipation skill as well.

4 Because deceptive actions involve conveying a false intention, the need to adapt coordination late in their unfolding action presents a challenging task to accurately produce the desired action outcome, to kick the ball accurately towards the corner of the goal during a penalty kick, for example.
